# The effectiveness of manual therapy applied to craniomandibular structures in the treatment of temporomandibular disorders: protocol for a systematic review

**DOI:** 10.1186/s13643-021-01623-7

**Published:** 2021-03-08

**Authors:** Giacomo Asquini, Alison Rushton, Laurent Pitance, Nicola Heneghan, Deborah Falla

**Affiliations:** 1 Centre of Precision Rehabilitation for Spinal Pain (CPR Spine), School of Sport, Exercise and Rehabilitation Sciences, College of Life and Environmental Sciences, University of Birmingham, Edgbaston, Birmingham, UK; 2 Institute of Experimental and Clinical Research, Health Sciences division, Neuro-Musculo-Skeletal-Lab (NMSK), Université Catholique de Louvain, Brussels, Belgium

**Keywords:** Temporomandibular disorder, Temporomandibular joint dysfunction syndrome, Pain, Manual therapy, Physical therapy, Temporomandibular joint, Masticatory muscles

## Abstract

**Background:**

The term temporomandibular disorder (TMD) includes disorders of the temporomandibular joints (TMJ), masticatory muscles and adjacent tissues. Several studies have examined the effectiveness of manual therapy (MT) for TMDs by evaluating changes in pain and maximum mouth opening (MMO). Nevertheless, the effectiveness of MT exclusively applied to the craniomandibular structures (craniomandibular manual therapy (CMMT)) on pain and TMJ range of motion remains unclear. This review aims to evaluate the effectiveness of CMMT on pain and TMJ range of motion in people with TMDs.

**Methods:**

This protocol is reported in line with the preferred reporting items for systematic reviews and meta-analysis protocols (PRISMA-P). Databases including MEDLINE, Embase, CINAHL, ZETOC, Web of Science, SCOPUS, PEDro, PubMed, Cochrane Library and Best Evidence, EBM reviews–Cochrane Central Register of Controlled Trials, Index to Chiropractic Literature ChiroAccess and Google Scholar will be searched from inception as well as key journals and grey literature. Randomised controlled trials involving adults with TMD that compare the effect of any type of CMMT (e.g. mobilisation) on pain and range of motion with a placebo intervention, controlled care intervention or other types of treatment will be included. Two reviewers will independently screen articles for inclusion, extract data, assess risk of bias (revised Cochrane risk of bias tool) for included studies and evaluate overall quality of evidence (Grading of Recommendations Assessment, Development and Evaluation). A meta-analysis will be conducted if possible. If not, a narrative synthesis will be conducted reporting the effectiveness of CMMT according to disorder type (TMJ disorders, masticatory muscle disorders and mixed disorders).

**Discussion:**

In this review, the effectiveness of MT applied to craniomandibular structures for the treatment of TMD will be evaluated. Results will be submitted for publication in a peer-reviewed journal and presented at conferences. We expect our findings will facilitate treatment planning for manual therapists managing patients with TMD and provide future clinical research implications.

**Systematic review registration:**

PROSPERO CRD42019160213

**Supplementary Information:**

The online version contains supplementary material available at 10.1186/s13643-021-01623-7.

## Background

The term temporomandibular disorder (TMD) encompasses disorders of the temporomandibular joints (TMJs), masticatory muscles and adjacent tissues [1]. The main characteristics of TMD are pain and limitations of jaw opening [2]. TMD is considered to be one of the primary causes of chronic orofacial pain with a significant impact on quality of life [3], and in developed countries, it remains a significant public health challenge [3, 4]. In Spain, the prevalence of TMD was reported to have increased from 8% in 1993 to 14% in 2015, which is in contrast with the general oral health improvement observed in the same period [5]. In addition to pain in the jaw region, patients with TMD often complain of pain in the neck and low back [4].

The multifactorial and often unclear aetiology of TMD has led to the development of numerous therapeutic interventions for the management of this painful disorder. Current clinical recommendations suggest a multidisciplinary approach with conservative interventions recommended for TMD [6]. Physical therapy (PT) is one of the most common treatments for the management of TMD [7], and it aims to reduce pain, increase joint mobility and correct aberrant motor behaviours [8, 9]. Within PT, manual therapy (MT) is commonly used given its positive effects on pain, muscle spasm and range of motion [9]. According to the American Academy of Orthopaedic Manual Physical Therapists (AAOMPT) Description of Advanced Specialty Practice (DASP), MT is “any hands-on treatment provided by the physical therapist” [10] (p.8). MT aims to enhance tissue extensibility, improve joint range of motion, mobilise or manipulate soft tissues and joints, produce relaxation, modulate pain and address problems with muscle activation and timing [11]. Randomised controlled trials (RCTs) have demonstrated the effectiveness of MT in patients with TMD by demonstrating changes in pain, maximum mouth opening (MMO) and pressure pain threshold (PPT) [12–15]. Different MT approaches have been investigated such as mobilisation of the TMJ [16], manipulation and mobilisation of the cervical spine [12, 13], soft tissue techniques and massage of masticatory and neck muscles [12, 14, 15].

In the last decade, systematic reviews and meta-analyses have examined the effectiveness of different PT interventions for TMD [17–22]. For example, Randhawa et al. [19] investigated the effectiveness of non-invasive interventions for TMD; however, no generalisable conclusions could be made regarding the effectiveness of MT since only one study was included. Paço et al. [18] examined the effectiveness of PT in the management of TMD but without separating different approaches such as manual techniques versus therapeutic exercise. Again, no conclusion on the effectiveness of MT alone could be drawn. One systematic review investigating the effectiveness of PT for TMD 17 included a sub-analysis by different treatments, but the small number of studies (< 10) with comparable interventions resulted in low levels of evidence for the effectiveness of MT. Only two systematic reviews [20, 21] have specifically examined the effectiveness of MT alone for TMDs, and these concluded that protocols of mixed MT show low levels of evidence for improving MMO and pain because of poor external validity, low methodological quality, heterogeneity of interventions and low internal validity of the included RCTs. It should be noted that Calixtre et al. [21] included articles with MT applied to different regions such as the craniomandibular area, cervical and thoracic spine, and Martins et al. [20] considered MT as “any manipulations of body tissues, muscles and bones by hands”. Including MT applied to remote sites likely influences the conclusions drawn.

There has been no systematic review specifically investigating the effectiveness of MT applied only to the craniomandibular structures (craniomandibular manual therapy (CMMT)) on pain and TMJ range of motion in TMDs; thus, we sought to evaluate the effectiveness of CMMT on pain and TMJ range of motion in people with TMD.

## Methods

The protocol is reported in line with the preferred reporting items for systematic review and meta-analysis protocols (PRISMA-P) checklist [23] (Additional file 1) and is registered in PROSPERO (CRD42019160213).

### Eligibility criteria

#### Inclusion criteria

##### Participants

Any trials that examined an adult population (>18 years of age) with the diagnosis of TMD in accordance with the Research Diagnostic Criteria for TMD (RDC/TMD) [24] or Diagnostic Criteria for TMD (DC/TMD) [25], or any trials with participants presenting signs and symptoms of TMD [1, 2, 26]. Please see the detailed diagnostic criteria listed within these original articles.

##### Outcome measures

The primary outcomes will be pain, and maximal mouth opening (MMO) since TMDs are principally characterised by pain and limitations of jaw opening. Pain will be defined as pain in the TMJ area and/or masticatory muscles, with possible irradiation to associated structures. MMO will be defined as the interincisal distance in millimetres measured during active MMO. The methods utilised to measure pain must be in line with recommendations of the Initiative on Methods, Measurement, and Pain Assessment in Clinical Trials (IMMPACT) [27] (e.g. visual analogue scale, numeric rating scale). The methods used to assess MMO must be in line with the DC/TMD clinical examination protocol (e.g. use of a ruler) [25].

##### Type of intervention

CMMT will be considered as “any hands-on treatment provided by the physical therapist” [10] (p.8) [as defined by the American Academy of Orthopaedic Manual Physical Therapists] targeted to TMJs, temporal muscles, masseter muscles, medial and lateral pterygoid muscles, suprahyoid muscles and other sites on the face and the head.

##### Study design

Any RCT comparing CMMT alone to a reference group not including CMMT (placebo intervention, controlled comparison intervention, standard care) will be considered. RCTs with multiple intervention groups will be included and approached based on the Cochrane Handbook for Systematic Reviews of Interventions [28].

##### Timing and setting

All time assessment points will be included and will be defined as immediate post-treatment, short-term (0–1 month), intermediate-term (2–6 months) and long-term follow-up (> 6 months). No restriction on setting and/or length of study intervention and follow-up will be applied in this review.

#### Exclusion criteria

If there is uncertainty that the CMMT intervention is directed to the craniomandibular area but involves other structures (e.g. neck, shoulder, trunk), the article will be excluded. Any trial or group combining CMMT with other interventions will be excluded. Articles including participants with previous surgery in the temporomandibular region, Eagle’s syndrome, rheumatic diseases and other severe comorbidities (e.g. fracture in the region, cancer, neurological disease) will be excluded. Articles which are not written in English will be excluded.

### Information sources

The search strategy will be performed from September to October 2020. It will be designed for each database by using medical subject headings (MESH) if available and relevant text words relating to TMD, TMJ, MT, PT and pain. The following electronic databases will be searched (from their inception onwards): MEDLINE (OVID interface), Embase (OVID interface), Scopus, Web of Science, CINAHL (EBSCO interface), PEDro, ZETOC, PubMed, Cochrane Library and Best Evidence, Index to Chiropractic Literature ChiroAccess, EBM reviews–Cochrane Central Register of Controlled Trials and Google Scholar.

Reference lists from included articles will be reviewed for additional potential studies. In addition, hand searching will be conducted in journals which commonly publish articles on the topics of MT and TMD, specifically *Musculoskeletal Science and Practice*, *Journal of Oral Rehabilitation*, *Physical Therapy*, *Clinical Rehabilitation*, *The Journal of Oral & Facial Pain and Headache*, *Journal of Manual and Manipulative Therapy*, *Journal of Applied Oral Science* and *The Clinical Journal of Pain*. Grey literature for unpublished research will cover British National bibliography for report literature, OpenGrey, dissertation abstracts and EThOS. Relevant authors in the field will be contacted to obtain information about unpublished or ongoing studies.

### Search strategy

A MEDLINE search strategy will be firstly planned and later adjusted for other databases. Syntax (truncation, wildcards and quotation marks) and operators will be revised based on the specific databases. The search strategy will combine terms and MESH about (1) TMD, (2) MT/PT and (3) RCT. The search process will be entirely completed online if possible. In the case of references not available online, a manual search will take place. If conference abstracts and proceedings are found during searching of grey literature, authors will be contacted. No date limits will be applied to guarantee the inclusion of all relevant articles. A draft search strategy for MEDLINE is provided in Additional file 2.

### Study records

#### Data management

All search results will be managed through EndNote; Endnote Version X8 (Clarivate Analytics) software.

#### Selection process

Two reviewers (GA/LP) will independently screen articles for inclusion by rating them as eligible/not eligible/unsure using the pre-defined eligibility criteria [29]. The eligibility criteria priority sequence is participants, study design, type of intervention, outcome measures and absence of exclusion criteria.

If an article cannot be excluded based on its title and abstract, it will be judged potentially relevant, and its full text will be examined [30]. If an article is ambiguous with regard to inclusion or exclusion, the full text will be examined [31]. Authors will be approached by email if required for clarity (a maximum of two attempts 1 week apart). Articles will be included if there is an agreement between both reviewers about the eligibility criteria. A third reviewer (DF) will arbitrate in the case of discrepancy of reviewers’ opinion following discussion [31]. The agreement between reviewers will be reported. A PRISMA flow diagram [23] will be used to present the included and excluded articles with reasons for exclusion.

#### Data collection process

A bespoke proforma based on the Cochrane form [28] will be designed and piloted to extract data from the included trials. Both reviewers will independently extract information. Any discrepancies between reviewers will be mediated by a third reviewer (DF). The data extraction form will be tested on five articles to enable reviewers to practise.

### Data items

Table 1 summarises the items that will be extracted from the included trials. Authors will be contacted for further information if necessary, as described above.

**Table 1:** Summary of items to be extracted from included trials

Content	Data items
Trial information	Authors, year of publication, location
Population	Sample size, type of TMD, inclusion/exclusion criteria
Intervention	Duration, frequency, detail of the type of manual therapy techniques
Comparison group	Type of comparison group
Outcome measures	Pain outcome measures MMO outcome measures
Follow-up assessment points	Detail of timing of follow-up assessments
Results	Between group differences at follow-up assessments

### Risk of bias in individual studies

The revised Cochrane risk-of-bias tool for randomised trials (RoB 2) [32] will be used to assess the risk of bias of the included articles since this tool is considered the best approach for RCTs [33]. Two independent reviewers (GA/LP) by following the full guidance document 32 edited by the ROB2 Development Group will evaluate and grade the risk of bias for all included studies. In the case of disagreements, a third reviewer (DF) will be consulted. Cohen’s *κ* will be utilised to estimate agreement between reviewers. The RoB 2 tool comprises five domains: bias arising from the randomisation process, bias due to deviations from intended interventions, bias due to missing outcome data, bias in measurement of the outcome, bias in selection of the reported result. Each domain consists of different questions to which there are five response options: yes, probably yes, probably no, no, no information. RoB 2 tool is hierarchically developed, so responses to questions furnish the basis for domain-level judgements about the risk of bias (low risk of bias, some concerns and high risk of bias) [32]. Likewise, these domain-level judgements provide the basis for an overall risk-of-bias judgement for the entire trial.

### Data synthesis

Data will be firstly synthesised with a qualitative synthesis. The type of TMD, assessment time points and outcomes of each study will be presented in tables.

From a quantitative perspective, the standardised mean difference (SMD) and 95% of the confidence intervals (CI) will be determined for MMO and pain. A SMD less than 0.5 will be considered as a small effect, a medium effect will be considered if SMD is from 0.5 to 0.8, and a SMD higher than 0.8 will be considered as a large effect [31]. A random-effects model will be used to produce a more prudent estimation of the real effect size of CMMT from the included studies [33].

Heterogeneity will be evaluated using the *I*^2^ statistical analysis (heterogeneity is defined as an *I*^2^ statistic ≥75%) [34]. If the included trials are homogeneous for outcomes and assessment points, a meta-analysis will be performed with pain and MMO as outcome data.

If a meta-analysis is not possible, a narrative synthesis will be conducted following the synthesis without meta-analysis (SWiM) in systematic review guidelines [35]. Data will be grouped according to outcome measures. If this grouping will not be possible, data will be arranged according to other variables (e.g. TMD type: TMJ disorders, masticatory muscle disorders and mixed disorders). Data will be presented in tables reporting key characteristics of the studies (e.g. study design, TMD type, sample size, assessment time points, comparator, RoB 2 and GRADE). Limitations of the synthesis methods and groupings used in this review will be discussed.

#### Additional analyses

We will perform a sub-group analysis of the outcomes where applicable. We will group studies according to the following variables: (1) assessment time point (e.g. immediate post-treatment, short-term [0–1 month], intermediate-term [2–6 months], and long-term follow-up [> 6 months]); (2) TMD type (e.g. TMJ disorders, masticatory muscle disorders and mixed disorders).

Sensitivity analysis will be performed to assess the robustness of the results by investigating the effects of including and excluding studies with high risk of bias.

### Meta-biases

Evaluation of possible reporting bias will be conducted through a search for unpublished studies, a further assessment of the consistency between protocols if available, trial registration and published articles included and evaluation of competing interests from different authors. Results will be narratively presented. Funnel plots will be generated if at least 10 studies are included [28]. The probability of study bias will be assessed by visual inspection.

### Confidence in cumulative evidence

The Grading of Recommendations, Assessment, Development and Evaluations (GRADE) approach will be used to assess the overall strength and quality of the evidence by following the GRADE Handbook [36]. This tool consists of five domains: risk of bias, inconsistency, indirectness, imprecision and publication bias; it ranges from high to very low quality of evidence.

### Patient and public involvement

The research question in this study was developed following consultations and discussion with patients. Patients will not be involved in the analysis and data collection of the systematic review.

## Discussion

TMD is a major public health concern and commonly presents as chronic orofacial pain. Altered processes of pain perception and psychological distress [3] could be considered as generic risk factors contributing to the onset and persistence of painful TMD which is characterised by pain experienced in the TMJ, masticatory muscles and associated structures [2]. Based on current knowledge of MT, it is known that hands-on techniques can induce an analgesic effect (e.g. pain modulation), affective responses (e.g. opioid and oxytocin activation) and increase joint range of motion [37].

Several systematic reviews and meta-analyses on the effectiveness of MT for TMD have been published [17–22]. Nevertheless, these reviews have examined the effectiveness of MT either without isolating specific techniques or without evaluating the effectiveness of MT applied specifically to the craniomandibular area. Even though MT is viewed as a comprehensive term including different techniques applied to different regions, the knowledge about the effectiveness of comparable techniques targeted to a specific region, i.e. craniomandibular structures, provides better guidance for clinicians using MT to manage people with TMD.

In the area of TMD management, there is the need for systematic reviews to support evidence-based practice [38], and as such, this systematic review will respond to this need by synthesising the current knowledge on the effectiveness of MT applied to the craniomandibular area in patients with TMD.

We will follow the Cochrane Handbook for Systematic Reviews of Interventions for any issue not discussed in this protocol [28]. Future findings of the review should be considered based on potential limitations at both study level (e.g. risk of bias dealing with randomization and assignment to intervention/control group, or to missing data) and review level (e.g. selection bias due to different diagnostic criteria for TMD, heterogeneity among studies due to differences in control/comparison intervention and assessment time point). Any amendments made to this protocol when conducting the study will be outlined in PROSPERO and in the final manuscript.

It is expected that the results of this systematic review will provide clinicians with the best possible evidence on the effectiveness, or not, of MT applied to craniomandibular structures in the treatment of people with TMDs.

## Supplementary Information

**Additional file 1.** PRISMA-P (Preferred Reporting Items for Systematic review and Meta-Analysis Protocols) 2015 checklist: recommended items to address in a systematic review protocol*.

**Additional file 2.** Draft search strategy for MEDLINE.

## Data Availability

Not applicable.

## Abbreviations

AAOMPT: American Academy of Orthopaedic Manual Physical Therapists
CMMT: Manual therapy applied to the craniomandibular structures
DC/TMD: Diagnostic Criteria for Temporomandibular Disorder
DASP: Description of Advanced Specialty Practice
CI: Confidence intervals
ESRC: Economic and Social Research Council
GRADE: Grading of Recommendations Assessment, Development and Evaluation
IMMPACT: Initiative on Methods, Measurement, and Pain Assessment in Clinical Trials
MESH: Medical subject headings
MMO: Maximum mouth opening
MT: Manual therapy
PRISMA-P: Preferred Reporting Items for Systematic review and Meta-Analysis Protocols checklist
PT: Physical therapy
RCT: Randomised controlled trials
RDC/TMD: Research Diagnostic Criteria for Temporomandibular Disorder
RoB 2: Revised Cochrane risk-of-bias tool for randomised trials
TMD: Temporomandibular disorder
TMJ: Temporomandibular joint
SDM: Standardised mean difference
