# Supplementary material for: The effectiveness of manual therapy applied to craniomandibular structures in the treatment of temporomandibular disorders: protocol for a systematic review
Source: Syst Rev. 2021 Mar 8;10:70. doi: 10.1186/s13643-021-01623-7 (PMC7941703; doi:10.1186/s13643-021-01623-7)
Supplement: Supplementary file 2 — Additional file 2. Draft search strategy for MEDLINE. [file 13643_2021_1623_MOESM2_ESM.docx]

Additional file 2: Draft search strategy for MEDLINE

| **BOX 1 - Draft search strategy for MEDLINE** | |
| --- | --- |
| 1. Temporomandibular disorders.mp 2. Temporomandibular disorder.mp 3. exp Temporomandibular Joint Disorders 4. Temporomandibular Joint Disorder.mp 5. exp Temporomandibular Joints 6. Temporomandibular Joint.mp 7. exp Craniomandibular Disorders 8. Craniomandibular Disorder.mp 9. exp Temporomandibular Joint Dysfunction Syndrome 10. Temporomandibular joint syndrome.mp 11. Temporomandibular Joint Dysfunction.mp 12. Temporomandibular Dysfunction.mp 13. TMD.mp 14. exp Facial Pain 15. TMJ.mp 16. exp Myofascial Pain Syndromes 17. Myofascial Pain Syndrome.mp 18. exp Masticatory Muscles 19. Masseter muscle.mp 20. exp Masseter muscles 21. Temporal muscle.mp 22. exp Temporal muscles 23. Myofascial Pain.mp 24. Orofacial Pain.mp 25. Craniomandibular pain.mp 26. Craniomandibular dysfunction.mp 27. Craniomandibular dysfunctions.mp 28. Jaw pain.mp 29. Jaw dysfunction.mp 30. Jaw dysfunctions.mp 31. Temporomandibular pain.mp 32. Temporomandibul*.mp 33. Craniomandibul*.mp 34. **1-33 OR** 35. exp Manipulation, Orthopedic 36. Manipulation.mp | 1. exp Manipulation, Spinal 2. exp Manipulation, Chiropractic 3. Spinal adjustment.mp 4. exp Manipulation, Osteopathic 5. exp Osteopathic Medicine 6. Osteopathic.mp 7. Orthopaedic*.mp 8. exp Orthopedics 9. Manipula* 10. exp Chiropractic 11. Chiropractic*.mp 12. Musculoskeletal therapy,mp 13. exp Musculoskeletal Manipulations 14. Manual therapy.mp 15. exp Physical Therapy Modalities 16. Physical therapy.mp 17. physiotherapy.mp 18. exp Rehabilitation 19. exp Physical and Rehabilitation Medicine 20. exp Mouth Rehabilitation 21. Physical Therap*.mp 22. Osteopathic*.mp 23. Manual ther*.mp 24. Physiotherap*.mp 25. **35-60 OR** 26. exp Randomised controlled trial 27. Randomised controlled trial.mp 28. exp Random Allocation 29. exp Clinical trial 30. exp Placebo 31. Trial*.mp 32. Group*.mp 33. Ramdom*.mp 34. **62-69 OR** 35. **34 AND 61 AND 70** |
